# Lean Mass and Body Fat Percentage Are Contradictory Predictors of Bone Mineral Density in Pre-Menopausal Pacific Island Women

**DOI:** 10.3390/nu8080470

**Published:** 2016-07-30

**Authors:** Maria Casale, Pamela R. von Hurst, Kathryn L. Beck, Sarah Shultz, Marlena C. Kruger, Wendy O’Brien, Cathryn A. Conlon, Rozanne Kruger

**Affiliations:** 1 Institute of Food Science and Technology, Massey University, Auckland 0632, New Zealand; nzmaria.78@gmail.com (M.C.); k.l.beck@massey.ac.nz (K.L.B.); m.c.kruger@massey.ac.nz (M.C.K.); w.j.obrien@massey.ac.nz (W.O.B.); C.conlon@massey.ac.nz (C.A.C.); r.kruger@massey.ac.nz (R.K.); 2 School of Sport and Exercise, Massey University, Wellington 6140, New Zealand; s.p.shultz@massey.ac.nz

**Keywords:** bone mineral density, Pacific Island, pre-menopausal, body composition, physical activity, dietary intake

## Abstract

Anecdotally, it is suggested that Pacific Island women have good bone mineral density (BMD) compared to other ethnicities; however, little evidence for this or for associated factors exists. This study aimed to explore associations between predictors of bone mineral density (BMD, g/cm^2^), in pre-menopausal Pacific Island women. Healthy pre-menopausal Pacific Island women (age 16–45 years) were recruited as part of the larger EXPLORE Study. Total body BMD and body composition were assessed using Dual X-ray Absorptiometry and air-displacement plethysmography (*n* = 83). A food frequency questionnaire (*n* = 56) and current bone-specific physical activity questionnaire (*n* = 59) were completed. Variables expected to be associated with BMD were applied to a hierarchical multiple regression analysis. Due to missing data, physical activity and dietary intake factors were considered only in simple correlations. Mean BMD was 1.1 ± 0.08 g/cm^2^. Bone-free, fat-free lean mass (LMO, 52.4 ± 6.9 kg) and age were positively associated with BMD, and percent body fat (38.4 ± 7.6) was inversely associated with BMD, explaining 37.7% of total variance. Lean mass was the strongest predictor of BMD, while many established contributors to bone health (calcium, physical activity, protein, and vitamin C) were not associated with BMD in this population, partly due to difficulty retrieving dietary data. This highlights the importance of physical activity and protein intake during any weight loss interventions to in order to minimise the loss of muscle mass, whilst maximizing loss of adipose tissue.

## 1. Introduction

There is little doubt that bone mineral density is a key component of healthy aging. The impact of osteoporotic fractures can be devastating, commonly resulting in the loss of mobility and independence, and increased mortality [1,2,3]. Maintenance of bone density throughout adulthood is crucial for reducing the risk of osteoporosis and associated fragility fractures in later life. Although approximately 70% of the variation in bone mineral density (BMD) is accounted for by genetics [4], individuals are able to maximise their bone health through a healthy body composition and lifestyle factors, such as dietary intake of calcium, protein, and vitamin C, adequate vitamin D status, and physical activity [5,6].

Body composition has a strong impact on bone health. While there is little doubt that total mass has an effect on bone, whether it is the effect of fat mass or lean mass that influences BMD is disputed. There are different mechanical and hormonal roles for muscle and fat: fat produces oestrogen, which has an established role in the maintenance of BMD—however, it has been suggested that serum oestrogen levels are independent of BMD [7,8]. There is also a possible role for the hormone leptin, with positive associations between circulating leptin and BMD observed [9,10,11]. Increased fat mass will also contribute to increased total mass, which in turn places a mechanical load on the bone and contributes to BMD [12,13]. In terms of lean mass, it is probable that if an individual has a high proportion of lean mass, they have engaged in a significant amount of load bearing activity, which stimulates bone growth [14]. Additionally, lean mass contributes to total mass, increasing the everyday load on bones. The effects of fat mass and lean mass on the maintenance of BMD also appears to depend on several variables: the skeletal site of measurement (regional vs. total body), indices used (i.e., *Z*-score, *T*-score, or BMD g/cm^2^, and whether adjusted for height or not), and menopausal status. From a clinical standpoint, none of these factors can be considered in isolation when investigating the true effect of body composition on bone.

Diet is a major modifiable lifestyle factor in bone health, with many different nutrients playing a role in BMD, including protein, minerals, fat-soluble and water-soluble vitamins. The primary materials required for the synthesis of extracellular bone are calcium, phosphorous, and protein [15]. Physical activity, in combination with dietary measures, is another important contributor to bone health. The mechanical loading associated with physical activity—specifically weight-bearing exercise—produces a strong osteogenic response [16].

While it is anecdotally suggested that Pacific Island women have good bone density and mass, there is little data examining this. Pacific Island people have one of the lowest rates of non-traumatic hip fracture in the world, further indicating good bone density and mass [17]. Pre- and post-menopausal Pacific Island women have a significantly higher bone mineral content (BMC) of the distal radius and ulna than European women [18], but it is not clear if data was adjusted for height and weight. However, controlling for body mass index (BMI) removed significant differences in a previous study examining the difference in bone mass in adults of different ethnicities [17].

The general assumption of greater BMD could be partially attributed to the average body size of Pacific Island women; total weight and lean mass have been shown to be significant determinants of bone density [19,20]. Similar findings were seen in Pacific Island children aged 3–7 years who have demonstrated a significantly greater bone density and bone mass compared to their age- and gender-matched European counterparts. However, this difference can be explained by greater height and weight, as when these factors are adjusted for, the differences in bone measures disappeared [21]. Conversely, the greater BMD in Pacific Island women seems to be less reliant on adequate nutrient intake, as dietary calcium intake in Pacific Island women is well below the recommended daily intake of 1000 mg/day, with a mean of 653 mg/day—lower than the 784 mg/day intake of the total population mean for New Zealand women [22]. Additionally, Pacific Island adults living in New Zealand are 2.3 times more likely to be deficient in vitamin D than non-Pacific Island adults [23], and dark skin colour is an acknowledged risk factor for low vitamin D status [24,25]. Physical activity could still play a role in bone health of Pacific Island women, as there has been an increase in levels of physical activity amongst this cohort over time. A 2003 Sport and Recreation New Zealand (SPARC) report shows that Pacific Island adults are less active than non-Pacific Island adults [26], while more recently the NZ National Health Survey data from 2014/2015 shows that with 46.2% of Pacific Island women meet the physical activity guidelines of at least 30 min of exercise five or more days per week [27].

Taken together, the aforementioned factors suggest genetic or ethnic differences in BMD, which warrant further exploration. The greatest health-related focus placed on Pacific Island people living in New Zealand concerns obesity and lifestyle-related diseases. Identification of the determinants of bone health in Pacific Island women could allow for greater refinement of health care interventions, minimising potential risk to bone. Additionally, the identification of BMD determinants in Pacific Island women contributes to an area lacking in research. The aim of this study was to explore the associations between body composition, nutrients, and physical activity with bone mineral density in pre-menopausal New Zealand-based Pacific Island women.

## 2. Materials and Methods

New Zealand European, Māori, and Pacific Island women were recruited for the women’s EXPLORE (Exploring Predictors Linking Obesity Related Elements) study [28], of which this present study is a part. Participants were included if they were aged between 16 and 45 years, post-menarcheal and pre-menopausal (defined by continual regular menstruation for one complete past year). Participants were excluded in the presence of pregnancy and lactation, low BMI (<18.5 kg/m^2^), chronic illness such as coronary heart disease, diabetes, cancer, gut disorders resulting in malabsorption, endocrine disorders, thyroid disease, kidney disease, liver disorders, blood-borne illnesses such as hepatitis, and dairy allergy. Participants were included in the Pacific Island group if they identified as Pacific Island ethnicity (with at least one parent also identifying as Pacific Islander).

Participants for this sub-study were recruited and screened during phase 1 of the women’s EXPLORE study [28]. Data required for this investigation were collected in the following two phases: During Phase 2, the testing phase, eligible participants visited Massey University’s Human Nutrition Research Unit, where total body composition, BMD, and dietary intake were measured. During Phase 3, participants completed a recent physical activity questionnaire (RPAQ) at home one week after phase 2 either online [29] or on paper, which was returned by post to the Human Nutrition Research Unit.

Total body BMD and BMC were measured using dual X-ray absorptiometry (DXA) (Discovery A, Hologic Inc., Marlborough, MA, USA). Body composition (total mass, body fat %, and lean mass) was calculated using air-displacement plethysmography via BodPod (Life Measurement Inc., Concord, CA, USA). The BodPod was used to measure body composition, as individuals with either very high or very low body fat % are not as accurately measured on the DXA as on the BodPod [30]. Total BMC was subtracted from lean mass to quantify fat-free, bone-free lean mass: this is represented by “lean mass” forthwith. Height was measured using a stadiometer (Holtain Ltd., Wales, UK). It was determined that BMD in g/cm^2^ was the most appropriate measure of bone to use in this population. The Writing Group for the International Society for Clinical Densitometry (ISCD) Position Development Conference have established that a *T*-score measure is only appropriate in individuals over the age of 50 [31]. *Z*-score is commonly used in pre-menopausal women to determine BMD, however this measure assumes the participants are age-matched, gender-matched, and ethnicity matched. The available ethnicities within the Hologic software database were not appropriate for this population; thus, a *Z*-score was not a reliable measure of BMD for this population.

Dietary data were gathered using a validated food frequency questionnaire (FFQ) [32], and physical activity data were collected via the RPAQ. Dietary data were analysed using FoodWorks 7 (Xyris Software Pty, Brisbane, Australia) to determine total energy, calcium, protein, and vitamin C intake. The researchers then transcribed RPAQ data into the current bone-specific physical activity questionnaire (cBPAQ) online interface [14] to obtain the cBPAQ score for each participant [14]. This score represents a scale, rather than a quantitative measure: the BPAQ focuses specifically on physical activity that is load bearing and has the capacity to affect bone, and as such, this score has been shown to correlate with BMD [14].

Statistical analyses were completed using SPSS (v.20, IBM Corporation, New York, NY, USA). All variables were tested for normality using the Shapiro–Wilk and Kolmogorov–Smirnov tests. Sample size was calculated using G*Power 3.1.9.2. It was calculated that a sample size of 77 participants was needed (80% power at a significance level of *p* < 0.05) to identify a medium effect size (*f*^2^ = 0.15) using three predictors of bone mineral density in a multiple linear regression analysis. Non-normal data was tested for homogeneity using the Levene’s test. If the data were shown to have a significant variance between groups using the Levene’s test, it was log-transformed and tested for normality again. Normally distributed data were reported as the mean ± standard deviation (SD), and non-normally distributed data were reported as the median (25th, 75th percentiles). Correlations were calculated using the Pearson’s test for normally distributed data and Spearman’s Rho for non-normally distributed data. A hierarchical block-wise multiple regression analysis was used to assess the ability of the independent variables to predict BMD (g/cm^2^), with the independent variable with the strongest correlation entered into the first block. Physical activity and calcium data were not included in the regression analyses, as missing data reduced the sample size to *n* = 43, which was insufficient for regression analyses with five independent variables. All assumptions for multiple regression analysis were met.

## 3. Results

There were 175 healthy Pacific Island women aged 16–45 years initially recruited for this study. Of these, five did not meet screening criteria, 25 declined to continue with the study, and 54 did not respond to invitations to participate post-screening. A total of 91 Pacific Island women took part in in the EXPLORE study. Of these, eight were unable to undergo a DXA scan due to equipment malfunction, leaving 83 suitable for inclusion in the multiple regression analysis used in this investigation. Of these, 24 did not return an RPAQ for assessment of physical activity. Twenty-seven women either under- or over-reported their dietary data when completing the FFQ (11 under-reported, 16 over-reported), as determined by the Goldberg equation using a physical activity level (PAL) of 1.5 as the cut-off point [33], and these data were removed from the dietary dataset. A total of 43 participants successfully completed all aspects of the study. Participant characteristics are shown in Table 1.

### 3.1. Body Composition and BMI

The mean total body weight was 90.4 ± 19 kg, and median BMI was 32.4 ± 6.8 kg/m^2^. The overall range of BMI scores are comparable to New Zealand-wide BMI scores of Pacific Island women, and are shown in Table 2.

### 3.2. Dietary Data

Overall, participants reported adequate intakes of calcium, protein, and vitamin C (Table 3), which is in disagreement with the 2008/2009 New Zealand Adult New Zealand Nutrition Survey (ANS) results. Specifically, this study cohort reported 1016 mg/day of calcium, compared to 653 mg/day reported in the ANS.

### 3.3. Physical Activity Data

The cBPAQ scores were not normally distributed, with the curve skewed to the left, indicating low overall physical activity. The median score was 1.7 (0.4, 5.2).

### 3.4. Correlations between Predictor Variables and BMD

Correlation co-efficients were calculated to identify any significant associations between predictor variables and BMD. Total mass, physical activity as measured by cBPAQ score, and calcium, protein, and vitamin C intake were excluded from the regression analysis (Table 4). Total mass was not entered into the regression due to high collinearity with both lean mass and body fat %. Physical activity and dietary factors reduced the number of full data sets to 43, contravening the requirements for adequate statistical power with multiple regression analysis [36].

**Figure 1:**
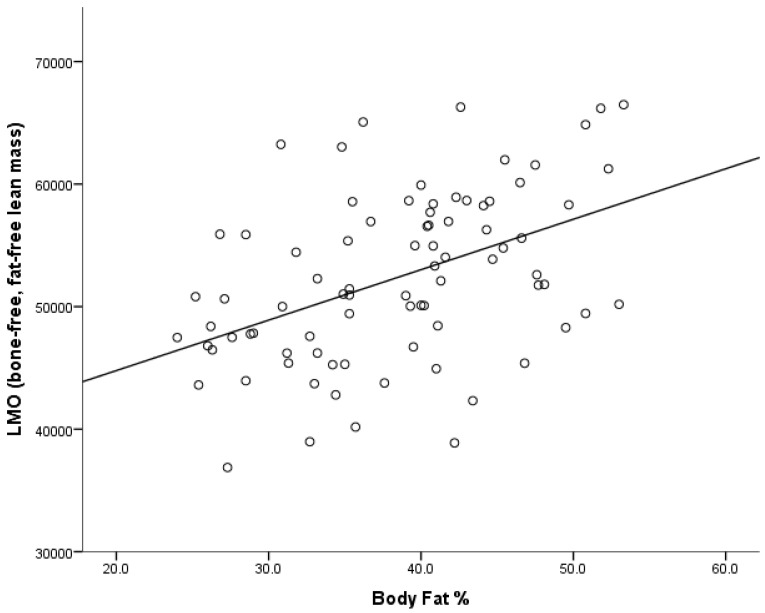
Association between lean mass and body fat %.

A significant, positive correlation (Figure 1) was also shown between lean mass and body fat % (*r* = 46, *n* = 83, *p* < 0.001); however, this relationship did not cause multicollinearity in the regression model.

### 3.5. Multiple Regression Analysis

A hierarchical multiple linear regression model was used to determine how much of the variance in BMD was explained by lean mass, age, and body fat % (Table 5). The multiple regression analysis shows that lean mass accounts for 21% of the variation in BMD (*F*(1, 82) = 21.5, *p* < 0.001). The introduction of age explains a further 5.5% of the variation (*F*(2, 82) = 14.4, *p <* 0.001). Finally, the addition of body fat % explains a further 13.5% of the variation (*F*(2, 82) = 15.3, *p* < 0.001). Together, lean mass, age, and body fat % explain 37.7% of the variability in BMD.

## 4. Discussion

The main purpose of this study was to explore associations of body composition, key nutrients, and physical activity with BMD in order to identify predictors of bone health in pre-menopausal Pacific Island women living in New Zealand. Lean mass, age, and body fat % were the only significant predictors of BMD, with no association shown between dietary calcium, protein, vitamin C, and physical activity with BMD.

It is probable that body composition plays an important role in bone health, with evidence for a role for lean and fat mass, as well as total mass. While there is currently no consensus as to what constitutes an appropriate threshold for defining high body fat % [37], the World Health Organisation defined obesity in women as ≥35% body fat [38]. However, the American Society of Bariatric Physicians published further guidelines indicating a threshold of ≥30% for women, which is used in most studies examining adiposity [39]. Based on this, the average body fat % of the participants in the current study is classified as high. However, it is well known that Pacific Island people are typically more muscular compared to most other ethnicities [40]. A study by Rush et al. [41] investigated the differences in body size and composition and found that Pacific Island women were significantly (*p* < 0.0001) more muscular than European, Māori, and Asian women. Furthermore, the fat-free mass (FFM) of the current study group (54.8 kg) was greater than that reported by Rush et al [41]. (46.4 kg), further supporting the role of lean mass as a predictor of bone health in Pacific Island women.

The present study shows a significant positive relationship between lean mass (that is, bone-free fat-free mass) and body fat %, but with accompanying low rates of physical activity. It is possible that the increase in non-contributory fat mass requires a subsequent increase in muscle mass to complete the activities of daily living. This suggests that the higher levels of total mass typically found in the Pacific Island body composition profile have a protective role for bone. The mechanism for this is that greater mass provides a greater mechanical loading on the skeleton, prompting the osteocytes to send a signal which either increases the activity of osteoblasts, or decreases the activity of osteoclasts [42]. Additionally, lean mass has a positive effect on BMD at specific load-bearing sites, such as the femoral neck and lumbar spine, but not at non-load bearing sites, such as the distal radius [43], where fat mass seems to have a stronger effect—potentially through metabolic action [44]. Given that the most common sites of fragility fractures are the lumbar spine and femoral neck [1], lean mass clearly has an important role in good bone health. Increased lean mass will also reduce the risk of falls and subsequent fractures in individuals.

In the current study, body fat % had an inverse relationship with BMD, suggesting that in these pre-menopausal Pacific Island women, increasing proportions of adiposity had a negative effect on bone health. This is supported by studies showing that fat mass correlates positively with bone health in post-menopausal, but not pre-menopausal, women [8,45,46,47,48,49,50,51]. However, the mechanism for this is yet to be elucidated. While the role of fat mass in post-menopausal women is undoubtedly mechanical, due to the increased load bearing it places on the skeleton, it is also thought to have a hormonal role due to the production of leptin and oestrogen by the adipocytes [11,52].

In other studies, the skeletal site of BMD measurement varies—whether the site of measurement is a load bearing site or not, and whether the scan is a total body measure or site-specific may affect the strength of body composition associations. Total body bone density appears to be affected by both lean mass and fat mass; however, this is likely to be dependent on menopausal status, with the most powerful association being between total lean mass and total body BMD in pre-menopausal women [47]. Additionally, there are strong correlations between lean mass and BMD at load bearing skeletal sites such as the femoral neck and lumbar spine, but not at non-load-bearing sites [43], such as the distal radius, where fat mass appears to have a stronger effect—potentially through metabolic action [44]. However, this finding has been contradicted by other studies, showing that fat mass significantly affected lumbar spine and femoral neck BMD in Chinese and Turkish women [49,50].

Although diet and physical activity variables were excluded from regression analyses due to under-powered sample size, the information did provide for comparisons to national databases. Reported calcium intake of the study group was higher (1016 mg per day) than that reported by the Adult Nutrition Survey [22] (653 mg per day) for New Zealand-wide Pacific Island women in the same age bracket. This higher intake of calcium may align with a slightly higher overall energy intake amongst participants, (average of 9334 kJ per day, compared to the NZ-wide Pacific Island average of 8318 kJ per day [22]), although the difference in energy consumption cannot solely explain the large discrepancy in calcium intake. Conversely, bone-specific physical activity shown by the cBPAQ amongst the participants was very low. The SPARC Physical Activity and Sport survey from 2003 [26] reports that 58% of Pacific Island women are considered to be active—defined as “took part in at least 2.5 h, but less than 5 h of sport/leisure-time physical activity in the 7 days before the interview” [26]. In the more recent 2014–2015 NZ National Health Survey, just 46.2% of Pacific women were reported as meeting the physical activity requirements of at least 30 min of physical activity on five or more days of the week [27]. It is likely that both of these figures represent greater physical activity than that reported by the cohort in the present study. However, given that the bone-specific physical activity questionnaire was used, this cannot be accurately determined.

Limitations of this study include the method of assessing physical activity: BPAQ is a bone-specific physical activity questionnaire, and has a current, past, and total component [14]. Due to the historical physical activity data not being available, only the current (cBPAQ) could be completed. The past BPAQ is shown to significantly correlate with bone strength at the heel for healthy young women [14], while the cBPAQ does not show a strong association in young women. The exclusion of the past BPAQ could account for the lack of association between physical activity and BMD. Including past BPAQ data in future studies would likely provide a much more complete picture of exactly how physical activity contributes to BMD. Another limitation was the use of bone-free, fat-free lean mass as a variable instead of appendicular skeletal muscle mass. Appendicular skeletal muscle mass is calculated by subtracting the total limb mass from the sum of wet bone mass (BMC divided by 0.55) and limb fat—a model which is thought appropriate as it is assumed that the weight of dermal tissue is negligible compared to skeletal muscle [53]. In terms of the study population, only 43 of the 83 participants successfully completed all facets of the study: this was due to some dietary data being excluded due to over- or under-reporting, and not all participants completing the physical activity questionnaire. A more general limitation to this study was that the testing session was not in close proximity to the majority of Pacific Island communities in Auckland. Many participants who were initially screened declined to continue participation, citing distance and travel time as a barrier. In future, research utilising portable equipment may encourage greater participant recruitment and retention.

## 5. Conclusions

Lean mass, age, and body fat percentage have significant, although contradictory, associations with BMD, with lean mass uniquely explaining 21% of the variance in BMD, age explaining 5.5%, and body fat percentage explaining 13.5%. This highlights the importance of healthcare professionals focusing on adequate protein intake during any weight loss interventions to minimise the loss of muscle mass, given that loss of lean mass has a negative effect on bone mass. There were no clear associations between BMD and physical activity, fat mass, age, or dietary calcium, protein, or vitamin C. The proportion of the variation in BMD that has remained unexplained (63.2%) could possibly be attributed to genetics [4]. Such variation could be the way that bone-specific nutrients are metabolised and utilised in bone turnover. Further exploration of bone metabolism, calcium absorption, and vitamin D status in Pacific Island women would provide insight to possible mechanistic differences in nutrient and bone metabolism that may account for the differences in BMD compared to other ethnicities.

## Figures and Tables

**Table 1:** Summary of participant characteristics.

Parameters	Participants (*n* = 83)	Range
Age (years)	28 (21, 37) *	16–45
Height (cm)	167.4 ± 5.8 ^†^	154.1–183.8
Weight (kg)	90.4 ± 19 ^†^	53.7–147.2
BMI (kg/m^2^)	32.4 ± 6.8 ^†^	20.4–54.9
Body fat %	38.4 ± 7.6 ^†^	24–53.3
Bone-free lean mass (kg)	52.4 ± 6.9 ^†^	36.9–66.4
Total body BMD (g/cm^2^)	1.2 ± 0.08 ^†^	0.93–1.29

* Median (25th percentile, 75th percentile); ^†^ Mean ± SD. BMD: bone mineral density.

**Table 2:** Comparison of proportions of BMI scores between study participants and NZ-wide Pacific Island women.

BMI Range (kg/m^2^)	Study Group	NZ-Wide [22]
18.5–24.9	13.2%	13.7%
25–29.9	28.6%	26.5%
≥30	58.2%	59.5%

Ministry of Health. (2014) [34]. Annual Update of Key Results 2013/2014: New Zealand Health Survey. Wellington: Ministry of Health.

**Table 3:** Daily intake of key nutrients for study participants and NZ-wide Pacific Island women.

Nutrient	Study Population (*n* = 56) Intake	NZ-Wide Intake (Adult Pacific Island Women) ^1^	Recommended Intake ^2^
Energy (kJ)	9334 (7210, 11,821) *	8318	BMR × PAL
Carbohydrate (% of total energy)	43 ± 8.2 ^†^	47	45%–65% of total energy intake
Fat (% of total energy)	35 ± 7 ^†^	35.2	20%–35% of total energy intake
Protein (% of total energy)	18 ± 3.8 ^†^	16.2	15%–25% of total energy intake
Protein (total g)	108.5 ± 42.4	81	0.8–1 g per kg of mass
Calcium (mg)	1016 ± 442 ^†^	653	14–18 years: 1300 mg/day
			19–50 years: 1000 mg/day
Vitamin C (mg)	125 (94, 216) *	99	14–18 years: 40 mg/day
			19–50 years: 45 mg/day

* Median (25th percentile, 75th percentile); ^†^ Mean ± SD; BMRxPAL – basal metabolic rate X physical activity level; ^1^ University of Otago, Ministry of Health. (2011). *A focus on Nutrition: Key findings of the 2008/2009 New Zealand Adult Nutrition Survey*. Wellington: Ministry of Health [22]; ^2^ National Health and Medical Research Council. (2006). *Nutrient Reference Values for Australia and New Zealand Including Recommended Dietary Intakes*. Canberra: NHMRC [35].

**Table 4:** Correlations of predictor variables with BMD.

Variables	*r*	*n*	*p*
Total mass	0.26	83	<0.05 *
cBPAQ	0.07	59	>0.05
Calcium	−0.04	56	>0.05
Protein	0.09	56	>0.05
Vitamin C	−0.01	56	>0.05

* Significant. cBPAQ: current bone-specific physical activity questionnaire.

**Table 5:** Hierarchical multiple regression showing predictors of bone mineral density.

B		SE B	95% CI B	Standar-dised β	*R*	*R*^2^	∆*R*^2^	*p*
Model 1					0.458	0.210	0.210 *	<0.001
Intercept	0.821	0.060	0.702, 0.941					
Lean mass	5.286 × 10^−6^	0.000	0.000, 0.000	0.458				
Model 2					0.514	0.265	0.055 **	<0.001
Intercept	0.763	0.063	0.637, 0.889					
Lean mass	5.258 × 10^−6^	0.000	0.000, 0.000	0.456				
Age	0.002	0.001	0.000, 0.004	0.234				
Model 3					0.632	0.377	0.135 ***	<0.001
Intercept	0.789	0.058	0.674, 0.889					
Lean mass	7.519 × 10^−6^	0.000	0.000, 0.000	0.651				
Age	0.003	0.001	0.002, 0.005	0.348				
Body Fat %	−0.005	0.001	−0.007, −0.002	−0.432				

Hierarchical block-wise enter technique. * *F*(1, 82) = 21.5; ** *F*(2,82) = 14.4; *** *F*(3, 82) = 15.32; B = beta standardized regression coefficient; SE = standard error of the coefficient

## Abbreviations

The following abbreviations are used in this manuscript:

ANS	Annual Nutrition Survey
BMC	Bone Mineral Content
BMD	Bone Mineral Density
BMI	Body Mass Index
BPAQ	Bone-specific Physical Activity Questionnaire
cBPAQ	Current Bone-specific Physical Activity Questionnaire
DXA	Dual X-ray Absorptiometry
FFM	Fat Free Mass
FFQ	Food Frequency Questionnaire
RPAQ	Recent Physical Activity Questionnaire
SPARC	Sport & Recreation New Zealand

## References

[1] Kanis J.A., Oden A., Johnell O., De Laet C., Jonsson B., Oglesby A.K. (2003). The components of excess mortality after hip fracture. Bone.

[2] Pasco J.A., Sanders K.M., Hoekstra F.M., Henry M.J., Nicholson G.C., Kotowicz M.A. (2005). The human cost of fracture. Osteoporos. Int..

[3] Taylor B.C., Schreiner P.J., Stone K.L., Fink H.A., Cummings S.R., Nevitt M.C., Bowman P.J., Ensrud K.E. (2004). Long-term prediction of incident hip fracture risk in elderly white women: Study of osteoporotic fractures. J. Am. Geriatr. Soc..

[4] Ralston S.H., de Crombrugghe B. (2006). Genetic regulation of bone mass and susceptibility to osteoporosis. Genes Dev..

[5] Rizzoli R. (2014). Nutritional aspects of bone health. Best Pract. Res. Clin. Endocrinol. Met..

[6] Wolff I., van Croonenborg J., Kemper H., Kostense P., Twisk J. (1999). The effect of exercise training programs on bone mass: A meta-analysis of published controlled trials in pre-and postmenopausal women. Osteoporos. Int..

[7] Lindsay R., Cosman F., Herrington B.S., Himmelstein S. (1992). Bone mass and body composition in normal women. J. Bone Miner. Res..

[8] Reid I.R., Ames R., Evans M.C., Sharpe S., France G.G.J.T., Lim T.M.T., Cundy T.F. (1992). Determinants of total body and regional bone mineral density in normal postmenopausal women—A key role for fat mass. J. Clin. Endocrinol. Met..

[9] Blain H., Vuillemin A., Guillemin F., Durant R., Hanesse B., De Talance N., Doucet B., Jeandel C. (2002). Serum leptin level is a predictor of bone mineral density in postmenopausal women. J. Clin. Endocrinol. Met..

[10] Pasco J.A., Henry M.J., Kotowicz M.A., Collier G.R., Ball M.J., Ugoni A.M., Nicholson G.C. (2001). Serum leptin levels are associated with bone mass in nonobese women. J. Clin. Endocrinol. Met..

[11] Thomas T., Burguera B., Melton L.J., Atkinson E.J., O'Fallon W.M., Riggs B.L., Khosla S. (2001). Role of serum leptin, insulin, and estrogen levels as potential mediators of the relationship between fat mass and bone mineral density in men versus women. Bone.

[12] Finkelstein J.S., Brockwell S.E., Mehta V., Greendale G.A., Sowers M.R., Ettinger B., Lo J.C., Johnston J.M., Cauley J.A., Danielson M.E. (2013). Bone mineral density changes during the menopause transition in a multiethnic cohort of women. J. Clin. Endocrinol. Met..

[13] MacInnis R.J., Cassar C., Nowson C.A., Paton L.M., Flicker L., Hopper J.L., Larkins R.G., Wark J.D. (2003). Determinants of bone density in 30-to 65-year-old women: A co-twin study. J. Bone Miner. Res..

[14] Weeks B.K., Beck B.R. (2008). The BPAQ: A bone-specific physical activity assessment instrument. Osteoporos. Int..

[15] Heaney R.P. (2000). Calcium, dairy products and osteoporosis. J. Am. Coll. Nutr..

[16] Rubin C.T., Lanyon L.E. (1984). Regulation of bone-formation by applied dynamic loads. J. Bone Jt. Surg. Am..

[17] Cundy T., Cornish J., Evans M.C., Gamble G., Stapleton J., Reid I.R. (1995). Sources of interracial variation in bone-mineral density. J. Bone Miner. Res..

[18] Reid I.R., Mackie M., Ibbertson H.K. (1986). Bone-mineral content in Polynesian and white New Zealand women. Br. Med. J..

[19] Bogl L.H., Latvala A., Kaprio J., Sovijarvi O., Rissanen A., Pietilainen K.H. (2011). An Investigation into the relationship between soft tissue body composition and bone mineral density in a young adult twin sample. J. Bone Miner. Res..

[20] Felson D.T., Zhang Y., Hannan M.T., Anderson J.J. (1993). Effects of weight and body mass index on bone mineral density in men and women: The Framingham study. J. Bone Miner. Res..

[21] Grant A.M., Gordon F.K., Ferguson E.L., Williams S.M., Henry T.E., Toafa V.M., Guthrie B.E., Goulding A. (2005). Do young New Zealand Pacific Island and European children differ in bone size or bone mineral?. Calcif. Tissue Int..

[22] University of Otago, Ministry of Health (2011). A Focus on Nutrition: Key Findings of the 2008/09 New Zealand Adult Nutrition Survey.

[23] Ministry of Health (2012). Vitamin D Status of New Zealand Adults: Findings from the 2008/09 New Zealand Adult Nutrition Survey.

[24] Chen T.C., Chimeh F., Lu Z., Mathieu J., Person K.S., Zhang A., Kohn N., Martinello S., Berkowitz R., Holick M.F. (2007). Factors that influence the cutaneous synthesis and dietary sources of vitamin D. Arch. Biochem. Biophys..

[25] Holick M.F., Maclaughlin J.A., Doppelt S.H. (1981). Regulation of cutaneous previtamin-D3 photosynthesis in man - skin pigment is not an essential regulator. Science.

[26] SPARC (2003). SPARC Facts: Results of the New Zealand Sport and Physical Activity Surveys (1997–2001).

[27] Ministry of Health (2015). Adult Data Tables: Health Status, Health Behaviours, and Risk Factors.

[28] Kruger R., Shultz S.P., McNaughton S.A., Russell A.P., Firestone R.T., George L., Beck K.L., Conlon C.A., von Hurst P.R., Breier B. (2015). Predictors and risks of body fat profiles in young New Zealand European, Māori and Pacific women: Study protocol for the women’s EXPLORE study. SpringerPlus.

[29] SurveyMonkey Inc.. https://www.surveymonkey.co.nz.

[30] Von Hurst P.R., Walsh D.C., Conlon C.A., Ingram M., Kruger R., Stonehouse W. (2015). Validity and reliability of bioelectrical impedance analysis to estimate body fat percentage against air displacement plethysmography and dual-energy X-ray absorptiometry. Nutr. Diet..

[31] The Writing Group for the ISCD Position Development Conferece (2004). Diagnosis of osteoporosis in men, premenopausal women, and children. J. Clin. Densitom..

[32] Houston Z.L. (2014). Development and Validation of a Semi-Quantitative Food Frequency Questionnaire to Assess Dietary Intake of Adult Women Living in New Zealand.

[33] Black A.E. (2000). Critical evaluation of energy intake using the Goldberg cut-off for energy intake: Basal metabolic rate. A practical guide to its calculation, use and limitations. Int. J. Obes. Relat. Metab. Disord..

[34] Ministry of Health (2014). Annual Update of Key Results 2013/14: New Zealand Health Survey.

[35] National Health and Medical Research Council (2006). Nutrient Reference Values for Australia and New Zealand Including Recommended Dietary Intakes.

[36] Green S.B. (1991). How many subjects does it take to do a regression analysis. Multivar. Behav. Res..

[37] Oliveros E., Somers V.K., Sochor O., Goel K., Lopez-Jimenez F. (2014). The concept of normal weight obesity. Prog. Cardiovasc. Dis..

[38] Vincenti M.A. (1996). Physical status: The use of and interpretation of anthropometry. J. Acad. Nutr. Diet..

[39] Okorodudu D., Jumean M., Montori V., Romero-Corral A., Somers V., Erwin P., Lopez-Jimenez F. (2010). Diagnostic performance of body mass index to identify obesity as defined by body adiposity: A systematic review and meta-analysis. Int. J. Obes..

[40] Rush E., Plank L., Chandu V., Laulu M., Simmons D., Swinburn B., Yajnik C. (2004). Body size, body composition, and fat distribution: A comparison of young New Zealand men of European, Pacific Island, and Asian Indian ethnicities. N. Z. Med. J..

[41] Rush E.C., Freitas I., Plank L.D. (2009). Body size, body composition and fat distribution: Comparative analysis of European, Maori, Pacific Island and Asian Indian adults. Br. J. Nutr..

[42] Seeman E., Delmas P.D. (2006). Bone quality—The material and structural basis of bone strength and fragility. N. Engl. J. Med..

[43] Harris S., Dallal G.E., Dawson-Hughes B. (1992). Influence of body weight on rates of change in bone density of the spine, hip, and radius in postmenopausal women. Calcif. Tissue Int..

[44] Glauber H.S., Vollmer W.M., Nevitt M.C., Ensrud K.E., Orwoll E.S. (1995). Body weight versus body fat distribution, adiposity, and frame size as predictors of bone density. J. Clin. Endocrinol. Metab..

[45] Baumgartner R.N., Stauber P.M., Koehler K.M., Romero L., Garry P.J. (1996). Associations of fat and muscle masses with bone mineral in elderly men and women. Am. J. Clin. Nutr..

[46] Compston J.E., Bhambhani M., Laskey M.A., Murphy S., Khaw K.T. (1992). Body-composition and bone mass in postmenopausal women. Clin. Endocrinol..

[47] Douchi T., Oki T., Nakamura S., Ijuin H., Yamamoto S., Nagata Y. (1997). The effect of body composition on bone density in pre- and postmenopausal women. Maturitas.

[48] Kirchengast S., Peterson B., Hauser G., Knogler W. (2001). Body composition characteristics are associated with the bone density of the proximal femur end in middle- and old-aged women and men. Maturitas.

[49] Liu S., Li J., Sheng Z., Wu X., Liao E. (2011). Relationship between body composition and age, menopause and its effects on bone mineral density at segmental regions in Central Southern Chinese postmenopausal elderly women with and without osteoporosis. Arch. Gerontol. Geriatr..

[50] Nur H., Toraman N.F., Arica Z., Sarier N., Samur A. (2013). The relationship between body composition and bone mineral density in postmenopausal Turkish women. Rheumatol. Int..

[51] Reid I., Evans M., Ames R. (1994). Volumetric bone density of the lumbar spine is related to fat mass but not lean mass in normal postmenopausal women. Osteoporos. Int..

[52] Thomas T., Gori F., Khosla S., Jensen M.D., Burguera B., Riggs B.L. (1999). Leptin acts on human marrow stromal cells to enhance differentiation to osteoblasts and to inhibit differentiation to adipocytes 1. Endocrinology.

[53] Heymsfield S.B., Smith R., Aulet M., Bensen B., Lichtman S., Wang J., Pierson R.N. (1990). Appendicular skeletal muscle mass: Measurement by dual-photon absorptiometry. Am. J. Clin. Nutr..

